# Response of soil respiration to experimental warming in a highland barley of the Tibet

**DOI:** 10.1186/s40064-016-1761-0

**Published:** 2016-02-20

**Authors:** Zhi-Ming Zhong, Zhen-Xi Shen, Gang Fu

**Affiliations:** Lhasa Plateau Ecosystem Research Station, Key Laboratory of Ecosystem Network Observation and Modeling, Institute of Geographic Sciences and Natural Resources Research, Chinese Academy of Sciences, Beijing, 100101 China

**Keywords:** Infrared radiator, Soil moisture, Temperature sensitivity, Tibetan Plateau, Warming magnitude

## Abstract

Highland barley is an important dominant crop in the Tibet and the croplands of the Tibet are experiencing obvious climatic warming. However, information about how soil respiration will respond to climatic warming in the highland barley system is still lacking. A field warming experiment using infrared heaters with two warming magnitudes was conducted in a highland barley system of the Tibet in May 2014. Five daily cycles of soil respiration was measured using a CO_2_ flux system (Li-8100, Li-COR Biosciences, Lincoln, NE, USA) during the period from early June to early September in 2014. The high and low experimental warming significantly increased soil temperature by 1.98 and 1.52 °C over the whole study period, respectively. The high experimental warming significantly decreased soil moisture. Soil respiration and its temperature sensitivity did not significantly change under both the high and low experimental warming. The response of soil respiration to experimental warming did not linearly correlate with warming magnitudes because a greater experimental warming resulted in a higher soil drying. Our findings suggested that clarifying the response of soil CO_2_ production and its temperature sensitivity to climatic warming need consider water availability in the highland barley system of the Tibet.

## Background

The global surface temperature is predicted to increase by 1.0–3.7 °C by the end of this century and the Tibetan Plateau, “the Third Pole of the Earth”, is one of the most sensitive regions to climatic warming (Fu et al. 2015; IPCC 2013). To better understand and predict the effect of such warming on alpine ecosystems, many warming experiments are performed; however, no studies are conducted in agricultural ecosystems on the Tibetan Plateau (Zhang et al. 2015). The croplands are experiencing obvious warming on the Tibetan Plateau (Shen et al. 2014) and about 80 % of the population lives in the cropland areas of the Tibet (Yang et al. 1996). These suggest that it remains unclear about how alpine agricultural ecosystems respond to future climatic warming on the Tibetan Plateau.

The Tibetan Plateau is one of the domestication centers of cultivated barley and the planting area of highland barley accounts for approximately 43 % of the grain crop area on the Tibetan Plateau (Dai et al. 2012; Zhao et al. 2015). Highland barley, the only crop which is grown at high altitudes, is one of the dominant crops on the Tibetan Plateau and is an important staple food of the Tibetan people (Wang et al. 2013). Highland barley can be made into a variety of conventional foods, such as fried noodles (i.e. zanba), wine, and health food due to its high beta-glucan (Zhang et al. 2002). The highland barley ecosystem is one sensitive system to climatic warming considering the significant relationship between the potential productivity of highland barley and air temperature (Zhao et al. 2015). However, no studies demonstrate the warming effects on highland barley ecosystem under controlled warming on the Tibetan Plateau. Therefore, it remains unclear about how highland barley system responds to future climatic warming on the Tibetan Plateau.

Soil respiration (*R*_*s*_) is one primary component of the carbon cycling in terrestrial ecosystems (Rustad et al. 2001). Soil temperature is one of the most important abiotic factors affecting *R*_*s*_ (Raich and Schlesinger 1992). Soil respiration generally increases exponentially with increasing soil temperature and the *Q*_10_ is often used to evaluate the temperature sensitivity of *R*_*s*_ (Lin et al. 2011; Raich and Schlesinger 1992). The response of *R*_*s*_ and its temperature sensitivity to climatic warming are different among different terrestrial ecosystems with regard to vegetation types and climatic conditions. For example, the effects of experimental warming on *R*_*s*_ are negative in a semi-arid grassland in Inner Mongolia, China (Liu et al. 2009), positive in a cropland at the southern Germany (Reth et al. 2009) or neutral in an alpine meadow in the Northern Tibet (Shen et al. 2015). The *Q*_10_ of *R*_*s*_ shows a negative correlation with experimental warming in a temperate agricultural ecosystem in Germany (Poll et al. 2013), positive in a subtropical cropland in China (Liu et al. 2012) or neutral in an alpine meadow in the Northern Tibet (Shen et al. 2015). Soil moisture (SM) is another one of the most important abiotic factors influencing *R*_*s*_, especially in arid and semi-arid ecosystems (Liu et al. 2009; Shen et al. 2015). SM can not only directly affect *R*_*s*_, but also influnence the *Q*_10_ of *R*_*s*_ (Lin et al. 2011; Shen et al. 2015). SM can also dampen the effect of soil temperature on *R*_*s*_ (Liu et al. 2009; Shen et al. 2015).

No studies discuss the response of *R*_*s*_ to climatic warming under controlled warming condition in agricultural ecosystems on the Tibetan Plateau. Therefore, it is still rudimentary about how climatic warming will affect *R*_*s*_ in the alpine croplands on the Tibetan Plateau. In this study, a field warming experiment using infrared heaters was conducted in a highland barley system of the Tibet. The main objective of this study was to analyze the response of *R*_*s*_ to warming.

## Methods

### Study area

The study area (91°21′E, 29°41′N, 3688 m above sea level) is located at the Lhasa Agro-ecosystem Research Station, Tibet Autonomous Region in China. Mean annual air temperature is 7.9 °C and mean annual precipitation is around 425 mm, with more than 90 % concentrated in the period from June to September (He et al. 2011). The annual air temperature was 8.5 °C and annual precipitation was 642.4 mm in 2014. That is, it is a warmer and wetter year (2014).

### Experimental design

The experimental soils have been used for crop planting since 1970s. Infrared heaters were used to increase temperature during the whole study period from May 26 to September 14 in 2014. There were three warming treatments with three replicates: the control (CK), low (1000 W) and high (2000 W) warming treatments with a total of nine 2 m × 2 m experimental plots. A 165 cm × 15 cm infrared heater (Kalglo Electronics Inc., Bethlehem, PA, USA) was suspended approximately 1.7 m above the ground in the center of each 2 m × 2 m plot. There was approximately 6–7 m distance between plots.

The highland barley was sown in May 26, 2014 and harvested in September 14, 2014. There was approximately 0.25 m between seeding rows and the seeding was 18.75 g m^−2^. There were no highland barley outside the 2 m × 2 m plot and there were no other vegetation types within each 2 m × 2 m plot. There were approximately 1150 plants per 2 m × 2 m plot.

### Soil temperature and soil moisture monitor

Soil temperature (*T*_*s*_) and SM sensors were set a depth of 0.05 m in the center of each plot. For each plot, the two sensors were connected to a data logger (HOBO weather station, Onset Computer, Bourne, MA, USA). The measurements were taken every minute, and the data was processed to provide an average every 5 min.

### Soil respiration measurements

A CO_2_ flux system (LI-8100, LI-COR Biosciences, Lincoln, NE, USA) with a 20 cm diameter opaque survey chamber was used to measure *R*_*s*_ (Fu et al. 2014). A polyvinyl chloride (PVC) collar (diameter, 20 cm; height, 5 cm) was inserted about 2–3 cm into the soil in the center of each plot (the soil temperature and soil moisture sensors were just beneath the PVC collar) in May 2014. The PVC collar was left the same place during the whole study period. The soil temperatures and moistures were consistent with those beneath the chambers. We started to measure *R*_*s*_ in May 2014. The opaque survey chamber was manually mounted on the PVC collar in each plot for the measurement of *R*_*s*_. Daily cycles of *R*_*s*_ measurements were generated from 8:00 to 8:00 on June 5–6 (approximately 3–4 days after sprouting), July 26–27 (approximately flag leaf stage), August 6–7 (approximately blooming stage), August 26–27 (approximately waxy ripeness stage) and September 6–7 (from yellow ripeness stage to full ripeness stage). The measuring interval was 2 and 3 h during the daytime (8:00–20:00) and nighttime (20:00–8:00), respectively.

### Statistical analysis

A repeated-measures ANOVA with experimental warming as the between subject factor and measuring date and time as the within subject factors was conducted for *R*_*s*_. Duncan multiple comparisons were performed among the three warming treatments.

Exponential regression analyses were conducted between *R*_*s*_ and *T*_*s*_, whereas linear regression analyses were conducted between *R*_*s*_ and SM for each treatment. For each treatment, a stepwise multiple regression analysis was used to analyze the relationships between *R*_*s*_ and *T*_*s*_ and SM, before which natural-logarithm transformations were made for *R*_*s*_ and SM.

We analyzed the sensitivity of *R*_*s*_ to soil temperature for each treatment using all measurement data according to

1 $$R_{s} = ae^{{bT_{s} }} ,$$

where *a* is the intercept of *R*_*s*_ when *T*_*s*_ is 0 °C (i.e. the *R*_*s*_ value when *T*_*s*_ is 0 °C), and *b* reflects the temperature sensitivity of *R*_*s*_ (Shen et al. 2015). The *b* values were used to calculate the respiration quotient (*Q*_10_)

2 $$Q_{10} = \frac{{R_{t + 10} }}{{R_{t} }} = e^{10b}$$

where *t* is a given reference soil temperature, *R*_*t*+10_ and *R*_*t*_ are the *R*_*s*_ values when soil temperature is *t* + 10 and *t* *°*C, respectively.

To decrease the disturbance of SM on *Q*_10_ of *R*_*s*_, we also chose data to compare the *Q*_10_ of *R*_*s*_ among the three treatments by the two following rules: (1) there were no correlations between *R*_*s*_ and *T*_*s*_ when SM <0.17 m^3^ m^−3^ for the high warming treatment. The *R*_*s*_ was obviously suppressed when SM <0.17 m^3^ m^−3^ for the low warming treatment. Therefore, only measuring data when SM >0.17 m^3^ m^−3^ were used; and (2) measuring date and time was consistent among the three treatments. That is, 26 groups of measured *R*_*s*_, *T*_*s*_ and SM were used for each treatment.

All the statistical analyses were performed using the SPSS software (version 16.0; SPSS Inc., Chicago, IL, USA).

## Results

**Fig. 1:**
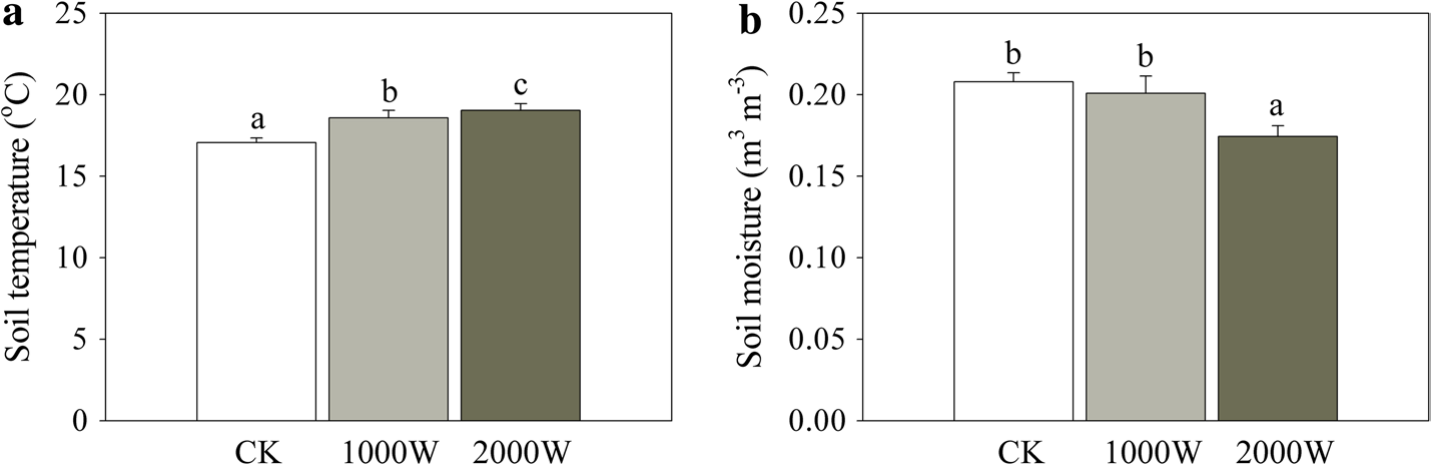
Effects of experimental warming on **a** soil temperature and **b** soil moisture over the whole study period in a highland barley of the Tibet. CK: the control, 1000 W: the low warming treatment, and 2000 W: the high warming treatment

Compared to the control, the low and high warming treatments significantly increased daily average *T*_*s*_ by 1.52 and 1.98 °C, respectively, over the whole study period (Fig. 1).The high warming treatment significantly decreased daily average SM by 16.1 % (−0.03 m^3^ m^−3^) over the whole study period (Fig. 1).

**Fig. 2:**
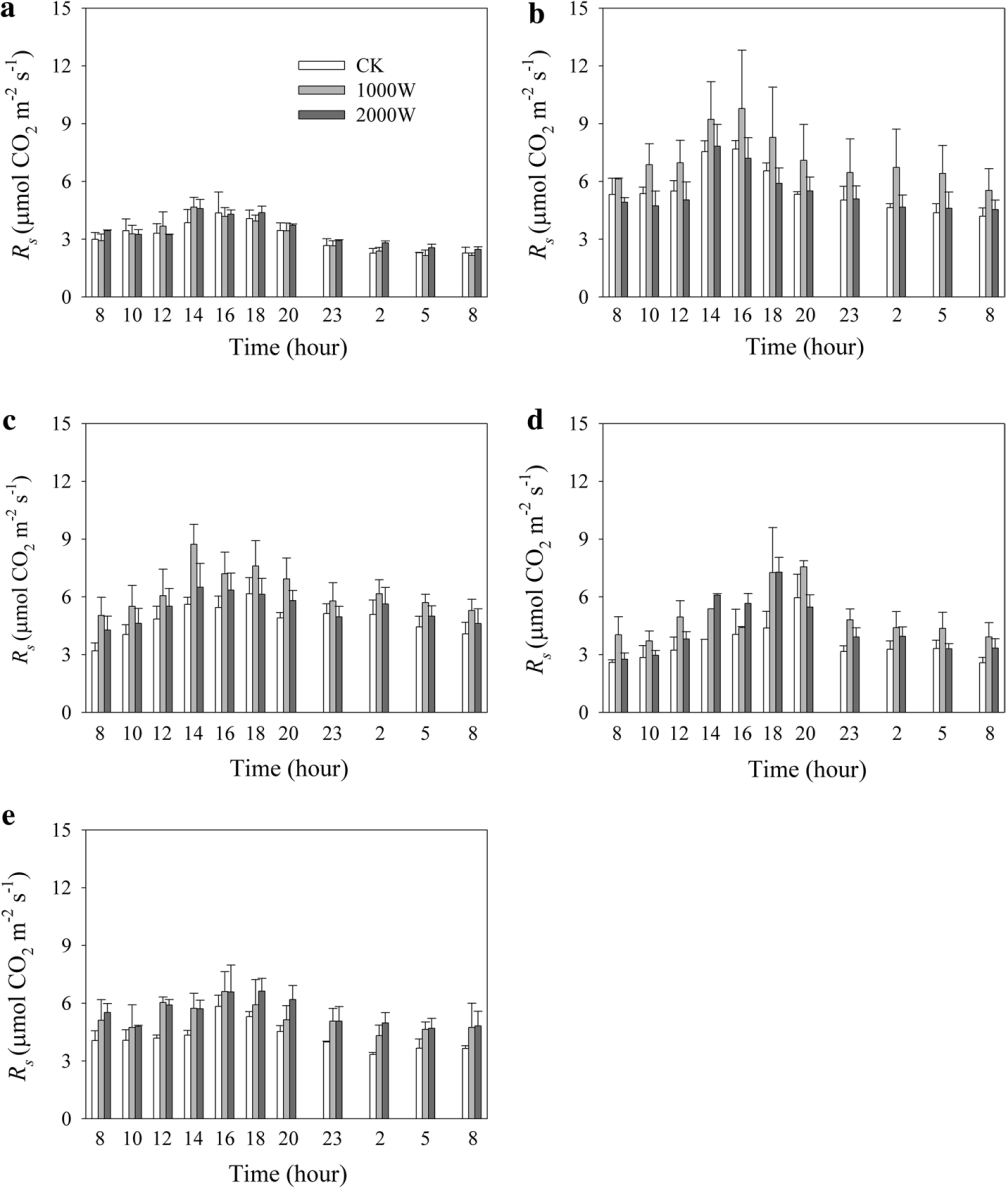
Effects of experimental warming on soil respiration (*R*_*s*_) in a highland barley of the Tibet (mean ± SE, *n* = 3). **a** June 5–6, **b** July 26–27, **c** August 6–7, **d** August 26–27, and **e** September 6–7 in 2014. CK: the control, 1000 W: the low warming treatment, and 2000 W: the high warming treatment

There were significantly temporal variations of *R*_*s*_ (Table 1; Fig. 2). The average *R*_*s*_ rates were 4.31, 5.41 and 4.85 µmol CO_2_ m^−2^ s^−1^ for the control, low and high warming treatments over the five measuring dates, respectively. There were no significant differences of *R*_*s*_ among the three warming treatments (Table 1).

**Table 1:** Repeated measures ANOVA for the main and interactive effects of experimental warming (W), measuring date (D) and time (T) on soil respiration (*R*_*s*_, µmol CO_2_ m^−2^ s^−1^) in a highland barley of the Tibet (*n* = 3)

Model	*df*	*F*	*p*
W	2, 6	0.99	0.43
D	4, 24	20.82	<0.001
T	10, 60	42.38	<0.001
W × D	8, 24	1.36	0.26
W × T	20, 60	0.57	0.72
D × T	40, 240	3.76	<0.05
W × D × T	80, 240	0.76	0.63

**Fig. 3:**
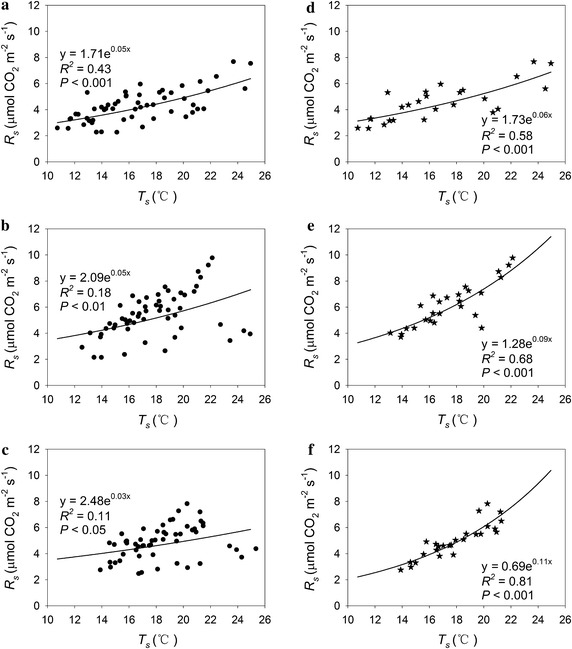
Relationship between soil respiration (*R*_*s*_) and soil temperature (*T*_*s*_) across all measuring data for **a** the control, **b** low warming treatment and **c** high warming treatment; and data included when soil moisture was >0.17 m^3^ m^−3^ and the measuring data and time were consistent among the three treatments for **d** the control, **e** low warming treatment and **f** high warming treatment

**Fig. 4:**
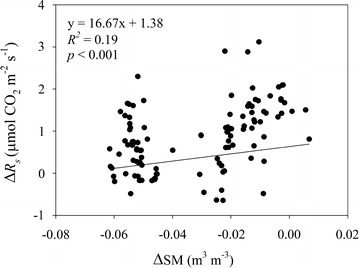
Relationship between the change of soil respiration (Δ*R*_*s*_) and the change of soil moisture (ΔSM) caused by the low and high warming treatment

Soil respiration significantly increased with increasing *T*_*s*_ (Fig. 3a–f). There was no significant effect of experimental warming on the apparent *Q*_10_ values when all the measuring data were used (control: 1.70; low warming treament: 1.65; high warming treament: 1.41). In contrast, there was significant effect of experimental warming on the *Q*_10_ of *R*_*s*_ when only the 26 groups data were used (*df* = 2, 75; *F* = 5.84; *p* < 0.01). In detail, the low and high warming treatments significantly increased the *Q*_10_ by approximately 0.67 and 1.22, while there was a negligible difference between the low and high warming treatments. The experimental warming-induced change of *R*_*s*_ significantly increased with that of SM (Fig. 4).

Stepwise regression analyses indicated that the variation of *R*_*s*_ was explained by *T*_*s*_ for the control plots, but explained by SM for the high warming treatment (Table 2). Soil temperature and SM together explained the variation of *R*_*s*_ for the low warming treatment (Table 2). However, the partial correlation between *R*_*s*_ and SM was larger than that between *R*_*s*_ and *T*_*s*_ for the low warming treatment (Table 2).

**Table 2:** Stepwise multiple regression analyses between soil respiration (*R*_*s*_) and soil temperature (*T*_*s*_) and soil moisture (SM), showing changes in the regression coefficient, significance probability (*p*), coefficient of determination (*R* ^*2*^) and partial correlation coefficient

Treatment	Coefficient	*R* ^2^	Partial correlation	*p*
CK				
Constant	0.54			<0.001
*T*_*s*_	0.05	0.43	0.65	<0.001
1000 W				
Constant	5.27			<0.001
*T*_*s*_	0.06	0.24	0.75	<0.001
SM	2.88	0.57	0.88	<0.001
2000 W				
Constant	5.43			<0.001
SM	2.20	0.39	0.62	<0.001

Natural logarithm transformations were made for *R*_*s*_ and SM prior to regression analysis. CK: the control, 1000 W: the low warming treatment, and 2000 W: the high warming treatment. *T*_*s*_ and SM coincided with *R*_*s*_

## Discussion

For the first time to our knowledge, this study quantified the warming effects on *R*_*s*_ and its temperature sensitivity in agricultural ecosystems of the Tibetan Plateau. Our finding that experimental warming did not significantly change *R*_*s*_ in this highland barley system was in line with our one previous study performed in alpine meadows of the Northern Tibet (Shen et al. 2015). However, there were significant positive responses of *R*_*s*_ to experimental warming in the alpine meadow of the Haibei station (Lin et al. 2011) and the Songpan County (Shi et al. 2012), an alpine steppe of the Northern Tibet (Lu et al. 2013) and forest ecosystems (Xu et al. 2010) on the Tibetan Plateau. These findings implied that the alpine ecosystems on the Tibetan Plateau did not always show a positive response of *R*_*s*_ to climatic warming. These results also indicated that the effect of climatic warming on *R*_*s*_ varied with not only ecosystems but also regions on the Tibetan Plateau. Compared to alpine grasslands and forests, the soil CO_2_ production of alpine agricultural ecosystem may have a lower response to climatic warming on the Tibetan Plateau. Our finding was also not in line with some previous studies which demonstrated that experimental warming significantly increased *R*_*s*_ in temperate and subtropical croplands (Liu et al. 2012; Reth et al. 2009). This phenomenon implied that alpine agricultural soil may not always have higher temperature sensitivity than temperate and subtropical agricultural soils.

In this study, the no significant response of *R*_*s*_ to experimental warming was most likely due to the experimental warming-induced soil drying (Table 2; Fig. 3). A meta-analysis showed that drying had a significant negative effect on *R*_*s*_ (Wu et al. 2011). Experimental warming-induced soil drying suppressed the effect of increased temperature on *R*_*s*_ in croplands (Poll et al. 2013; Wall et al. 2013), alpine and temperate grasslands (Liu et al. 2009; Shen et al. 2015). Soil drying can suppress soil microbial activity and microbial respiration (Fu et al. 2012; Liu et al. 2009), and plant photosynthesis and primary production (Fu et al. 2013; Xu et al. 2013), all of which are positively correlated with *R*_*s*_ (Fu et al. 2014; Iqbal et al. 2010). The finding that the effect of experimental warming on *R*_*s*_ did not correlate with warming magnitudes may be attributed to the finding that a higher experimental warming resulted in a greater soil drying in our study system.

Different from many previous studies which exhibited experimental warming significantly decreased temperature sensitivity of soil respiration (Luo et al. 2001; Poll et al. 2013; Suseela and Dukes 2013; Zhou et al. 2012), experimental warming did not significantly affect the temperature sensitivity of *R*_*s*_ across all the measuring data in this highland barley system (Fig. 3a–c). In contrast, when the disturbance of SM was dampened (i.e. when SM >0.17 m^3^ m^−3^), experimental warming significantly increased the temperature sensitivity of *R*_*s*_ in this highland barley system (Fig. 3d–f). Soil respiration did not significantly correlate with soil temperature when SM was smaller than 0.17 m^3^ m^−3^ for the high warming treatment. Similarly, the temperature sensitivity of *R*_*s*_ declined with drought in a New England old-field ecosystem (Suseela and Dukes 2013) and  increased with increasing SM in an alpine meadow in the Northern Tibet (Shen et al. 2015). Therefore, soil water availability affected the temperature sensitivity of soil respiration and the negligible response of temperature sensitivity derived from all the measuring data may be due to soil drying caused by experimental warming.

## Conclusions

Experimental warming-induced soil drying masked the effect of increased soil temperature on soil respiration in the highland barely system. Water availability will be a limited factor for the carbon emission of alpine agricultural soil under climatic warming on the Tibetan Plateau. The alpine soil may not show a positive feedback to climatic warming, which contrasted with the finding as most of previous studies have suggested on the Tibetan Plateau.

## References

[CR1] Dai F, Nevo E, Wu DZ, Comadran J, Zhou MX, Qiu L, Chen ZH, Beiles A, Chen GX, Zhang GP (2012). Tibet is one of the centers of domestication of cultivated barley. Proc Natl Acad Sci.

[CR2] Fu G, Shen Z, Zhang X, Zhou Y (2012). Response of soil microbial biomass to short-term experimental warming in alpine meadow on the Tibetan Plateau. Appl Soil Ecol.

[CR3] Fu G, Zhang X, Zhang Y, Shi P, Li Y, Zhou Y, Yang P, Shen Z (2013). Experimental warming does not enhance gross primary production and above-ground biomass in the alpine meadow of Tibet. J Appl Remote Sens.

[CR4] Fu G, Zhang X-Z, Zhou Y-T, Yu C-Q, Shen Z-X (2014). Partitioning sources of ecosystem and soil respiration in an alpine meadow of Tibet Plateau using regression method. Pol J Ecol.

[CR5] Fu G, Shen ZX, Sun W, Zhong ZM, Zhang XZ, Zhou YT (2015). A meta-analysis of the effects of experimental warming on plant physiology and growth on the Tibetan Plateau. J Plant Growth Regul.

[CR6] He YT, Sun W, Zhang ZX, Shi PL, Qu YC, Zhong ZM, Hu J, Zhao HZ (2011). Effect of organic/inorganic compound fertilizer on the yield of crop and fodder double-cropping system in the Tibetan Plateau. Chin J Eco-Agric.

[CR7] Stocker TF, Qin D, Plattner G-K, Tignor M, Allen SK, Boschung J, Nauels A, Xia Y, Bex V, Midgley PM, IPCC (2013). Summary for policymakers. Climate change 2013: the physical science basis. Contribution of working group I to the fifth assessment report of the intergovernmental panel on climate change.

[CR8] Iqbal J, Hu RG, Feng ML, Lin S, Malghani S, Ali IM (2010). Microbial biomass, and dissolved organic carbon and nitrogen strongly affect soil respiration in different land uses: a case study at Three Gorges Reservoir Area, South China. Agric Ecosyst Environ.

[CR9] Lin XW, Zhang ZH, Wang SP, Hu YG, Xu GP, Luo CY, Chang XF, Duan JC, Lin QY, Xu B, Wang YF, Zhao XQ, Xie ZB (2011). Response of ecosystem respiration to warming and grazing during the growing seasons in the alpine meadow on the Tibetan plateau. Agric For Meteorol.

[CR10] Liu WX, Zhang Z, Wan SQ (2009). Predominant role of water in regulating soil and microbial respiration and their responses to climate change in a semiarid grassland. Glob Chang Biol.

[CR11] Liu Y, Chen ST, Hu ZH, Ren JQ, Shen XH (2012). Effects of simulated warming on soil respiration in a cropland under winter wheat-soybean rotation. Environ Sci.

[CR12] Lu XY, Fan JH, Yan Y, Wang XD (2013). Responses of soil CO_2_ fluxes to short-term experimental warming in alpine steppe ecosystem, Northern Tibet. PLoS One.

[CR13] Luo YQ, Wan SQ, Hui DF, Wallace LL (2001). Acclimatization of soil respiration to warming in a tall grass prairie. Nature.

[CR14] Poll C, Marhan S, Back F, Niklaus PA, Kandeler E (2013). Field-scale manipulation of soil temperature and precipitation change soil CO_2_ flux in a temperate agricultural ecosystem. Agric Ecosyst Environ.

[CR15] Raich JW, Schlesinger WH (1992). The global carbon dioxide flux in soil respiration and its relationship to vegetation and climate. Tellus Ser B Chem Phys Meteorol.

[CR16] Reth S, Graf W, Reichstein M, Munch JC (2009) Sustained stimulation of soil respiration after 10 years of experimental warming. Environ Res Lett 4. doi:10.1088/1748-9326/4/2/024005

[CR17] Rustad LE, Campbell JL, Marion GM, Norby RJ, Mitchell MJ, Hartley AE, Cornelissen JHC, Gurevitch J, Gcte N (2001). A meta-analysis of the response of soil respiration, net nitrogen mineralization, and aboveground plant growth to experimental ecosystem warming. Oecologia.

[CR18] Shen ZX, Fu G, Yu CQ, Sun W, Zhang XZ (2014). Relationship between the growing season maximum enhanced vegetation index and climatic factors on the Tibetan Plateau. Remote Sens.

[CR19] Shen ZX, Li YL, Fu G (2015). Response of soil respiration to short-term experimental warming and precipitation pulses over the growing season in an alpine meadow on the Northern Tibet. Appl Soil Ecol.

[CR21] Shi FS, Chen H, Chen HF, Wu Y, Wu N (2012). The combined effects of warming and drying suppress CO_2_ and N_2_O emission rates in an alpine meadow of the eastern Tibetan Plateau. Ecol Res.

[CR22] Suseela V, Dukes JS (2013). The responses of soil and rhizosphere respiration to simulated climatic changes vary by season. Ecology.

[CR23] Wall GW, McLain JET, Kimball BA, White JW, Ottman MJ, Garcia RL (2013). Infrared warming affects intrarow soil carbon dioxide efflux during vegetative growth of spring wheat. Agron J.

[CR24] Wang J, Li HR, Li YH, Yu JP, Yang LS, Feng FJ, Chen Z (2013). Speciation, distribution, and bioavailability of soil selenium in the Tibetan Plateau kashin-beck disease area-a case study in Songpan County, Sichuan Province, China. Biol Trace Elem Res.

[CR25] Wu ZT, Dijkstra P, Koch GW, Penuelas J, Hungate BA (2011). Responses of terrestrial ecosystems to temperature and precipitation change: a meta-analysis of experimental manipulation. Glob Chang Biol.

[CR26] Xu ZF, Wan CA, Xiong P, Tang Z, Hu R, Cao G, Liu Q (2010). Initial responses of soil CO_2_ efflux and C, N pools to experimental warming in two contrasting forest ecosystems, Eastern Tibetan Plateau, China. Plant Soil.

[CR27] Xu WF, Yuan WP, Dong WJ, Xia JZ, Liu D, Chen Y (2013). A meta-analysis of the response of soil moisture to experimental warming. Environ Res Lett.

[CR28] Yang GH, Du ES, Xu ZY, Wang CF (1996). Productivity of land resources and population carrying capacity in Xizang.

[CR29] Zhang GP, Wang JM, Chen JX (2002). Analysis of beta-glucan content in barley cultivars from different locations of China. Food Chem.

[CR30] Zhang XZ, Shen ZX, Fu G (2015). A meta-analysis of the effects of experimental warming on soil carbon and nitrogen dynamics on the Tibetan Plateau. Appl Soil Ecol.

[CR31] Zhao XY, Wang WJ, Wan WY, Li H (2015). Influence of climate change on potential productivity of naked barley in the Tibetan Plateau in the past 50 years. Chin J Eco-Agric.

[CR32] Zhou JZ, Xue K, Xie JP, Deng Y, Wu LY, Cheng XH, Fei SF, Deng SP, He ZL, Van Nostrand JD, Luo YQ (2012). Microbial mediation of carbon-cycle feedbacks to climate warming. Nat Clim Chang.

